# Charged Adsorbates on Metallic Surfaces from Periodic to Open Boundary Conditions

**DOI:** 10.1021/acs.jpcc.5c01216

**Published:** 2025-05-26

**Authors:** Nicéphore Bonnet, Nicola Marzari

**Affiliations:** Theory and Simulation of Materials (THEOS), Ecole Polytechnique Fédérale de Lausanne, Lausanne 1015, Switzerland

## Abstract

Understanding the thermodynamics of adsorbates on surfaces is central to many (electro)­catalysis applications. In first-principles calculations, additional challenges arise when considering charged adsorbates owing to long-range electrostatic interactions in the in-plane and normal directions. Here, we derive an analytical correction to obtain the energy profiles of individual charged adsorbates on metallic surfaces from finite-cell calculations in periodic boundary conditions. The method is illustrated by calculating the adsorption energy profiles of Li^+^, Na^+^, and K^+^ on graphite from first-principles, highlighting the very slow convergence with system size of the periodic calculations and the need to correctly recover the infinite limit.

## Graphical abstract

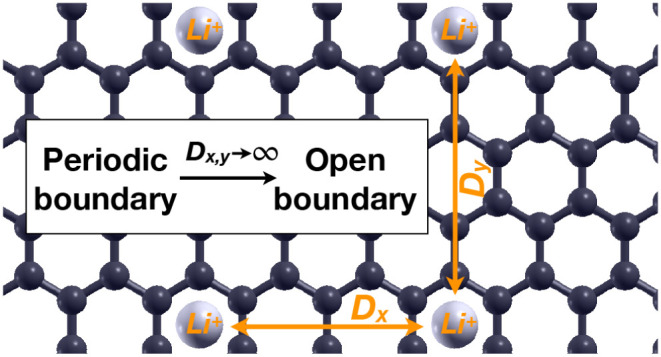

## Introduction

Charged species at surfaces and interfaces play an important role in many scientific and technological developments, including (opto)­electronics, electrochemistry, corrosion, and chemical sensing. First-principles simulations of such processes are often performed in periodic-boundary conditions (PBC), making them subject to the size-dependence of long-range electrostatics. Correction schemes must then be introduced to remove the effect of periodic images and recover the properties of the system in open-boundary conditions (OBC), that is, of the charged species isolated at the interface.^1–14^ Longitudinal corrections are now routinely included in the self-consistent calculation of slab systems representing the interface.^2–4^ In the context of electrochemistry, they have also been applied a posteriori—that is, after the electronic structure calculation—in the form of capacitive energy corrections.^15,16^ Lateral corrections (i.e., parallel to the interface) have presented some additional challenges, translating into several approaches. In a first approach, the OBC energy is obtained as the infinite-cell energy extrapolated from a series of finite-cell calculations typically via polynomial scaling expressions.^17–19^ However, C. Freysoldt et al. have highlighted the limitations of this kind of empirical approach in comparison with more physics-based extrapolation schemes.^5^ Consequently, a second approach consists in calculating a posteriori energy corrections based on an electrostatic surrogate model of long-range interactions in the system. The surrogate model consists mainly in specifying the free charge distribution representing the charged species of interest, and the spatial profile of the dielectric permittivity, often simplified as longitudinal dependences of the longitudinal and transverse components of the effective permittivity, ε_⊥_(*z*) and ε_∥_(*z*).^6–8^ The resulting electrostatic model is solved directly from the Poisson equation^6,9^ or from the image-charge method,^8^ and then used for extrapolating finite-cell results to the infinite cell size, or directly for obtaining the difference between PBC and OBC energies. Finally, a third approach is to include the correction scheme directly in the self-consistent calculation of the electronic structure. This has been done notably by truncating the Coulombic interaction,^10–12^ or by adding a self-consistent correction potential obtained from the same kind of surrogate model as described above.^13^ Importantly, this third approach has the advantage of ensuring the correct OBC charge density distribution, at the expense of modifying the electronic-structure calculation code.

In this paper, an a posteriori correction scheme is performed analytically for the case of charged adsorbates on metallic surfaces. The calculations here are performed in vacuum, but dielectric screening from solvents can also be readily included.^20,21^ The approach is used in combination with the effective screening medium (ESM) method^2^ to first generate a set of finite-cell energies in longitudinal OBC. The analytical solution of an electrostatic surrogate model is then used to fit the energy scaling behavior vs lateral cell dimensions, and in particular to obtain the OBC limit.

The paper is organized as follows. First, the motivation of calculating adsorption energy profiles of charged species on metallic surfaces is discussed in the typical context of electrocatalysis and electron-transfer reactions. The present analytical correction scheme is then outlined, and illustrated with Li^+^ adsorption on platinum. Finally, the approach is applied to the adsorption of ionic and neutral lithium, sodium and potassium on graphite surfaces, a topic of interest notably for lithium-ion and next-generation sodium-ion batteries.^22^ In this case, the validity of the metal surrogate model underlying the correction scheme is shown to rely on the transverse dielectric response of the surface graphene layer. The results are compared with finite-cell results from preexisting literature, and the significance of the lateral correction is discussed in comparison with the purely capacitive longitudinal correction. Notably, capacitively corrected energies are shown to converge slowly with the system size, with adsorption energy differences of the order of 1 eV between the 6 × 3 and 1000 × 1000 cells, hence the importance of recovering the appropriate infinite-cell limit from the lateral correction.

## Methodology

### Charged Adsorbates on Metallic Surfaces

Let us consider the generic electrochemical reaction on an electrode surface,

1 $$A_{\infty }^{+}+e^{-}\to A_{\#}$$

where $A_{\infty }^{+}$ is an ion in solution, electrosorbing to the neutral state *A*_#_ on the surface. The absolute charge of the electron *e* ^–^ will be denoted as *e*. As illustrated in Figure 1, the system *S* is defined as the electrode covered with N adsorbates *A*_#_ and bearing a net charge *q* in units of *e*. This system is connected to an external potentiostat of electropotential $\Phi_{p}$ , and to the electrolyte reservoir of $A_{\infty }^{+}$ ions of chemical potential $\mu_{A_{\infty }^{+}}$ . Denoting the canonical free energy of *S* as *G*(*N*,*q*), its grand-canonical free energy is then $\mathrm{G̅}\left(N,q\right)=G\left(N,q\right)-N\mu_{A_{\infty }^{+}}+\left(Ne-q\right)\Phi_{p}$ . The thermodynamic equilibrium relations ${\frac{\partial \mathrm{G̅}}{\partial N}}_{|q}={\frac{\partial \mathrm{G̅}}{\partial q}}_{|N}=0$ then imply

2 $$\mu_{A_{\#}}\equiv {\frac{\partial G}{\partial N}}_{|q}=\mu_{A_{\infty }^{+}}-e\Phi_{p}$$

and $\Phi \equiv {\frac{\partial G}{\partial q}}_{|N}=\Phi_{p}$ . eq 2 is the classical expression of the Nernst equation.^23^ Importantly, the chemical potential $\mu_{A_{\#}}$ is here defined in the canonical ensemble (fixed electrode charge *q*), while an alternative expression is found in refs 24 and 25 using chemical potentials defined in the grand-canonical ensemble (fixed electropotential). We can also decompose ${\frac{\partial G}{\partial N}}_{|q}$ as [*G*(*N*+1,*q*+1) - *G*(*N*,*q*)] + [*G*(*N*+1,*q*) - *G*(*N*+1,*q*+1)]. The first term can be defined as the chemical potential of the ion on the surface at a fixed total charge *q* + 1 of the {electrode + ion} system, denoted as $\mu_{A_{\#}^{+}}$ , and for a macroscopic electrode, the second term effectively equals $-e{\frac{\partial G}{\partial q}}_{|N}$ and thus $-e\Phi_{p}$ . Injecting the resulting relationship, $\mu_{A_{\#}}=\mu_{A_{\#}^{+}}-e\Phi_{p}$ , into eq 2, we obtain the equivalent, although less common, expression of the Nernst equation $\mu_{A_{\#}^{+}}=\mu_{A_{\infty }^{+}}$ .

**Figure 1:**
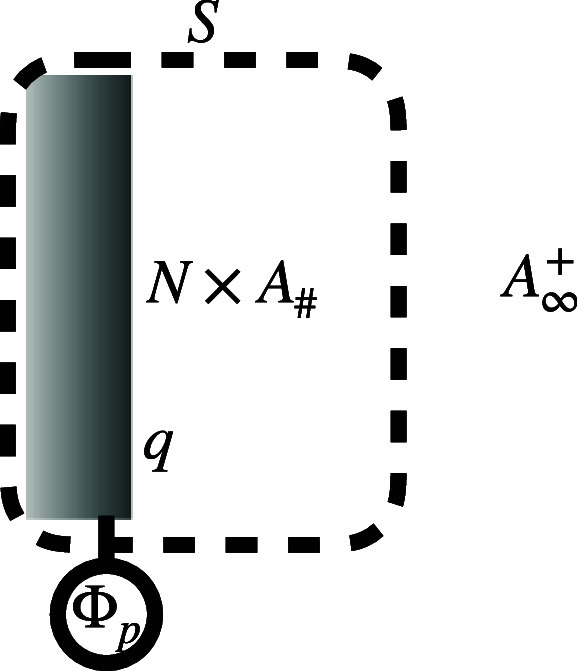
Electrochemical system *S* consisting of the electrode covered with *N* electrosorbates *A*_#_ and bearing a net charge *q*. This system is connected to an external potentiostat of electropotential $\Phi_{p}$ , and to the electrolyte reservoir of $A_{\infty }^{+}$ ions of chemical potential $\mu_{A_{\infty }^{+}}$ .

Similarly, electron transfer dynamics are determined, under the adiabatic Born–Oppenheimer condition,^23^ by the free energy landscape $g_{A^{+}}\left(r,\nu \right)$ of the {electrode + ion} system as a function of the coordinates *r* of the ion and ν of the surrounding solvent, at a fixed total charge of the system. At each point of the coordinate space, the actual redox state of the ion depends on the ground-state electronic charge density present on the ion.

Accordingly, methods to obtain the free energy profile of a charged adsorbate in the vicinity of an electrode at a constant total charge are of fundamental interest for the study of electrocatalytic reactions.^24,25^ Specifically, the present paper proposes an analytical correction to obtain OBC energies from PBC energies of an ion on a metallic surface.

### Correction Scheme

The methodology is illustrated with the case of a Li^+^ ion adsorbing on a hollow site of a Pt (111) surface, represented as a fixed, 3-layer slab (Figure 2). The lateral dimensions of the orthorhombic cell are *N*_*x*_ × 2.77 Å and *N*_*y*_ × 4.80 Å, where *N*_*x*_ and *N*_*y*_ are integer numbers. The constant total explicit charge of the cell (+1) is compensated by introducing an implicit ESM counter-charge (−1) at a distance *z*_*ESM*_, such that the overall system is neutral. The total energy *E* ^*tot*^(*z*) of the system is calculated with density-functional theory (DFT) in PBC as a function of the Li^+^ longitudinal coordinate *z* referenced to the position of the slab outer layer. DFT calculations are performed with Quantum ESPRESSO,^26^ using the Perdew–Burke–Ernzerhof (PBE) exchange correlation functional^27^ in combination with pseudopotentials of the SSSP PBE efficiency 1.1.2 library^28^ for ionic cores. We use kinetic energy cutoffs of 45 and 360 Ry for wave functions and the charge density, respectively, a 6 × 6 × 1 k-point mesh, and a Gaussian smearing with a broadening parameter of 0.01 Ry, for converging relative energies within 10 meV. We note that, by construction, the ESM scheme removes the influence of longitudinal periodic images, thus reducing the actual periodicity from three dimensions (3D-PBC) to two dimensions (2D-PBC). The following method is thus used to further remove the energy from the ESM electrode and from lateral interactions.

**Figure 2:**
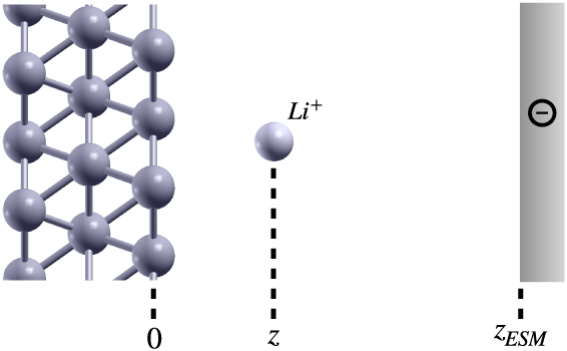
Slab setup for the adsorption of the ion on the electrode surface, with the ESM electrode bearing an implicit neutralizing countercharge.

The ion adsorption energy profile *E*_*ads*_(*z*) is obtained in the finite cell as

3 $$E_{ads}\left(z\right)=E^{tot}\left(z\right)-E_{slab}^{tot}-E_{Li^{+}}^{tot}$$

where $E_{slab}^{tot}$ and $E_{Li^{+}}^{tot}$ are the total energies of the neutral slab and of the isolated Li^+^ ion in vacuum, respectively. The energy $E_{Li^{+}}^{tot}$ is obtained by using the “parabolic” OBC electrostatic correction of Dabo et al.^3^ implemented in the ENVIRON package for first-principles electrochemistry.^21^ The dependence of *E*_*ads*_(*z*) on the finite cell size is isolated by splitting the energy into

4 $$E_{ads}\left(z\right)=E_{loc}\left(z\right)+E_{capa}\left(z\right)+E_{el}\left(z\right)$$

where *E*_*loc*_(*z*) contains the local (chemical + short-range electrostatic) interactions, expected to converge quickly as a function of the cell size, *E*_*capa*_(*z*) is the longitudinal capacitive energy, and *E*_*el*_(*z*) contains the remaining long-range electrostatic energy, including lateral periodic-image interactions. The capacitive energy is here given, in atomic units (a.u.), by $E_{capa}\left(z\right)=\left[\left(1-\xi \right)\left(\Phi \left(z\right)-\Phi_{0}\right)+\frac{4\pi }{\Omega }\xi \left(z_{ESM}-z\right)\right]/2$ , where ξ is the (a priori *z*-dependent) net charge on the ion (in units of *e*), Ω is the transverse surface area of the cell, −*e*Φ­(*z*) is the Fermi energy as a function of the ion position, and Φ_0_ is the work function of the bare slab. Moreover, taking a two-dimensional Gaussian-charge array above an ideal metal surface as an electrostatic surrogate model of the system, *E*_*el*_(*z*) is approximated, in a.u., as^2^

5 $$E_{el}\left(z\right)∼{Ẽ}_{el}\left(z\right)=-\xi^{2}\left(\frac{\eta }{\sqrt{\pi }}+\frac{\sqrt{\pi }}{\Omega \eta }\right)+\frac{\xi^{2}\pi }{\Omega }\underset{g\neq 0}{\sum }\frac{1}{g}\left[\mathrm{erfc}\left(\frac{g}{2\eta }\right)-\exp\left[2g\left(z-z_{1}\right)\right]\right]$$

where $\frac{1}{\eta \sqrt{2}}$ is the standard deviation of the Gaussian distribution (here, η = 1.67 bohr^–1^ as optimized by the ESM code),^2^ and *z*_1_ is the position of the ideal metal surface. In the second term, the sum is taken over the nonzero lateral reciprocal vectors **g** of norm *g*. Finally, the ion charge can here be found from the electrostatic relationship, Ω­(Φ­(*z*) – Φ_0_) = (1 – ξ)/*C*(*z*) + 4π­(*z_ESM_* – *z*), where *C*(*z*) is the surface capacitance between the slab and the ion plane. In the present case, we find ξ = 1 at all positions, implying $E_{capa}\left(z\right)=\frac{e\left(\Phi \left(z\right)-\Phi_{0}\right)}{2}$ . From now on, the adsorption energy profile will designate the longitudinally corrected quantity *E*(*z*) = *E*_*ads*_(*z*) – *E*_*capa*_(*z*).

Then, in line with eq 4, the estimate of the local energy *Ẽ*_*loc*_(*z*) is obtained as the mean of *E*(*z*) – *Ẽ*_*el*_(*z*) over different cell sizes. This, in turn, allows for defining the model adsorption energy profile as *Ẽ*(*z*) = *Ẽ*_*loc*_(*z*) + *Ẽ*_*el*_(*z*), which can be extrapolated to arbitrary cell sizes based on the analytical expression, eq 5, for *Ẽ*_*el*_(*z*). Figure 3 shows the results of this procedure, where the value of *z*_1_ = 1.45 Å has been used to minimize the mean error between the calculated *E*(*z*) and model *Ẽ*(*z*) energies. The calculated (diamonds) and model (dashed curves) adsorption energy profiles are in good agreement, with a mean absolute error smaller than 0.02 eV for all positions over the calculated cell sizes. The OBC adsorption energy profile (continuous curve) is obtained as the model profile *Ẽ*(*z*) converged in the large 1000 × 1000 cell.

**Figure 3:**
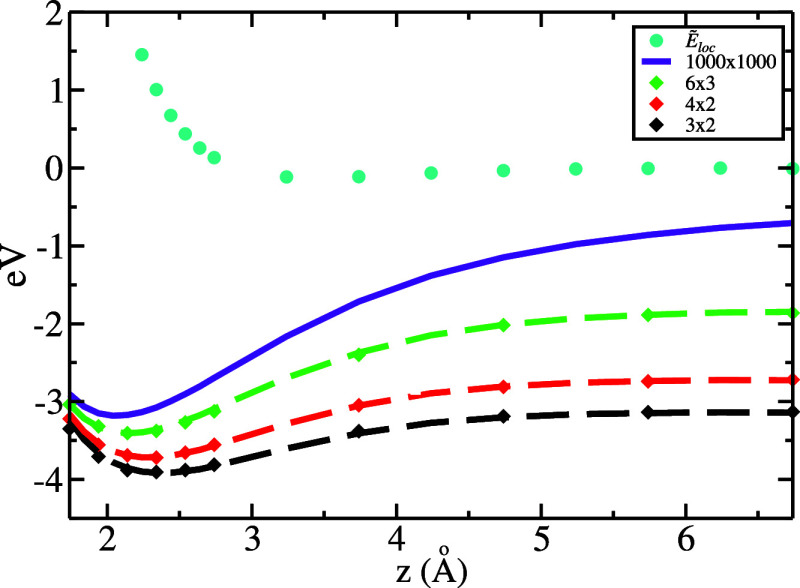
Longitudinal energy profiles of Li^+^ adsorbing on a hollow site of Pt(111): adsorption energy profiles *E*(*z*) obtained from DFT calculations (diamonds) and *Ẽ*(*z*) from the model in finite cells (dashed curves) and in the infinite-cell (OBC) limit (continuous curve), and the local energy profile *Ẽ*_*loc*_(*z*) (dots). As expected, the OBC adsorption energy profile converges to 0 at infinite distances (not shown here).

## Results and Discussion

The methodology is applied to the adsorption of Li^+^, Na^+^ and K^+^ on a hollow site of graphite. The lateral dimensions of the orthorhombic cell are *N*_*x*_ × 2.46 Å and *N*_*y*_ × 4.26 Å. In the following, the ion position and energy at the minimum of the adsorption energy profile will be simply referred to as the adsorption position (*z*_*ads*_) and energy. To simplify the analysis and isolate lateral scaling effects, the adsorption energy profiles are obtained, unless stated otherwise, at fixed, unrelaxed positions of the graphite slab.

As on platinum, the ion charges are equal to +1 at all positions. Energy profiles are found to be similar on graphene and multilayer graphite within 0.01 eV. Detailed results of the fitting procedure are shown for Li^+^ in Figure 4a, where the optimal value of *z*_1_ = 0.88 Å is found to correspond to the plane of charge density accumulation when adding a small electronic charge to the slab. The mean absolute difference (error) between the calculated and model energy profiles over the different cell sizes is lower than 0.02 eV at the adsorption position (∼1.7 Å) and beyond, while increasing more rapidly as the ion gets closer to the slab. This latter degradation manifests the increasing crudeness of the ideal metal approximation of the surrogate model at short distances. The local energy and OBC adsorption energy profiles of Li^+^, Na^+^ and K^+^ are reported in Figure 4-b, with corresponding adsorption energies of −3.01 eV, −2.20 eV and −1.77 eV, respectively. For information, relaxing the graphite positions shifts the Li^+^ adsorption energy by −0.19 eV on graphene and by −0.06 eV on multilayer graphite in the 3 × 2 cell.

**Figure 4:**
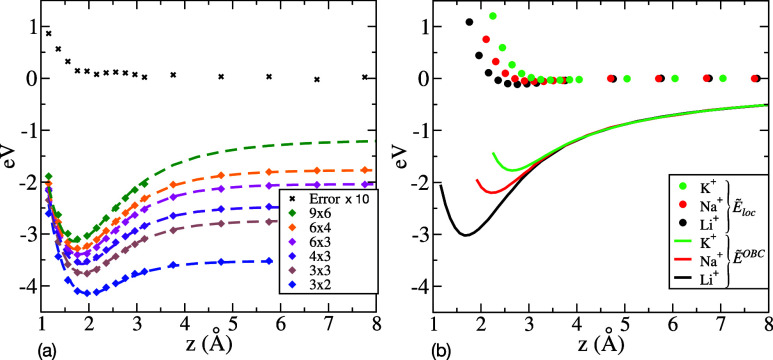
(a) Detailed results of the fitting procedure for Li^+^ adsorbing on a hollow site of graphite. The calculated *E*(*z*) (diamonds) and model *Ẽ*(*z*) (dashed curves) PBC adsorption energy profiles are shown in different cell sizes. For legibility, the mean absolute deviation (error) between calculated and model energies is reported with a 10× magnification. (b) Local energy *Ẽ*_*loc*_(*z*) (dots) and OBC adsorption energy *Ẽ* ^*OBC*^(*z*) profiles (continuous curves) for Li^+^, Na^+^, and K^+^ on the hollow site. The OBC adsorption profiles are obtained by extrapolating the model to the 1000 × 1000 cell, and they are equivalent to the image-charge interaction plus the local energy.

A chemical analysis shows almost no hybridization between the graphite and ion electronic states, explaining the fact that *Ẽ*_*loc*_(*z*) ≳ 0 at all distances. The overall adsorption energy profile is thus mainly the sum of the attractive image-charge interaction and of a repulsive electrostatic interaction in the short-range. The onset of the latter is driven by the electronic spread of the ion, hence the Li^+^ < Na^+^ < K^+^ ordering of adsorption positions and energies.

Interestingly, the scaling behavior of the system energy is well reproduced by the metal surrogate model despite the low dielectric permittivity (∼3) of graphene in the longitudinal direction.^29^ The underlying mechanism of this behavior is 2-fold. First, the ion and the ESM countercharge generate a net zero average field on the slab, thus effectively not interacting with the bulk permittivity. Accordingly, the electronic response of the slab is mainly confined within the upper surface of the outer graphene layer as shown in the left of Figure 5. Second, the calculated charge density difference induced on the graphite surface by the presence of the ion is similar to that of the electrostatic surrogate model (right side of Figure 5). Therefore, the validity of the latter relies on the metallic response of the π-orbital plane of graphene in the lateral direction.

**Figure 5:**
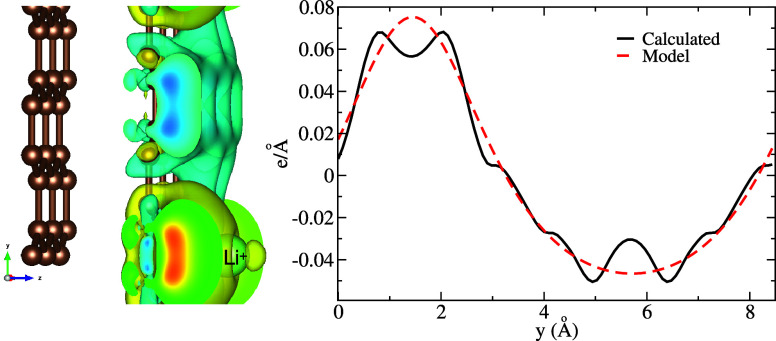
Charge density difference induced on graphite by the presence of the ion, here Li^+^ at *z* = 2.76 Å in the 3 × 2 cell. Left side: Three-dimensional view of the calculated charge density difference. Right side: Calculated and model linear charge density differences along the *y*-axis.

For comparison, adsorption energies obtained by using only the longitudinal capacitive correction are reported as a function of the cell size in Figure 6. In method 1, the adsorption energy is obtained directly as *E*(*z*_*ads*_), that is, referencing the adsorbed state to Li^+^ isolated in vacuum as implied by eq 3. Alternatively, in method 2, it is obtained as *E*(*z*_*ads*_) – *E*(+∞), that is, referencing the adsorbed state to the Li^+^ array in vacuum. In method 1, cell sizes larger than 9 × 6 are necessary to converge the adsorption energy within 0.1 eV of the OBC limit.

**Figure 6:**
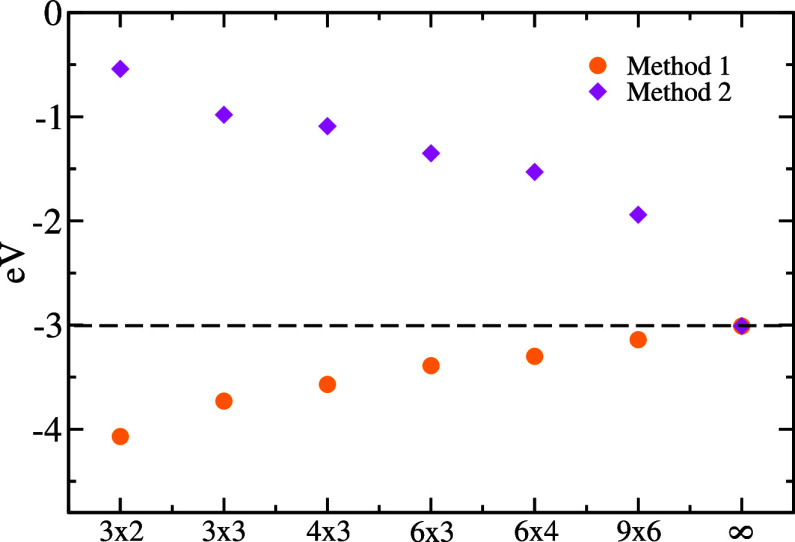
Li^+^ adsorption energies obtained by using only the capacitive correction, when referencing the adsorbed state to the isolated (method 1) or periodically repeated (method 2) Li^+^ in vacuum.

Additionally, the adsorption energy of the neutral Li^0^ atom can also be obtained as

6 $$E_{0}\left(z_{ads}\right)=E_{0}^{tot}\left(z_{ads}\right)-E_{slab}^{tot}-E_{Li^{0}}^{tot}$$

where the total energy $E_{0}^{tot}$ is now calculated at a zero explicit charge of the {slab + Li^0^} system, and $E_{Li^{0}}^{tot}$ is the total energy of Li^0^ isolated in vacuum. In contrast to the ionic case, the binding energy is found to be slightly smaller on graphene than on multilayer graphite, here by 0.08 eV in the 3 × 2 cell, in agreement with the value of 0.1 eV found by Valencia et al. in a cell of comparable size.^30^ The calculated adsorption energy on multilayer graphite is −1.22, −1.41, and −1.53 eV in the 3 × 2, 4 × 3, and 6 × 4 cell, respectively, in good agreement with previous values of {−1.18, −1.10}, −1.39 and −1.59 eV calculated with PBE in cells of comparable sizes.^30,31^ For information, relaxing the graphite positions shifts the adsorption energy by 0.02 eV in the 3 × 2 cell. The neutral adsorption energy can be related to the ionic one by rewriting (implicitly at *z*_*ads*_)­

7 $$E_{0}=\left(E_{0}^{tot}-E_{c}^{tot}\right)+\left(E_{c}^{tot}-E_{slab}^{tot}-E_{Li^{+}}^{tot}\right)+\left(E_{Li^{+}}^{tot}-E_{Li^{0}}^{tot}\right)=\Delta E_{c}^{tot}+E+IE_{Li}$$

where $IE_{Li}=E_{Li^{+}}^{tot}-E_{Li^{0}}^{tot}$ is the lithium ionization energy, $E_{c}^{tot}=E^{tot}-E_{capa}$ , and $\Delta E_{c}^{tot}=E_{0}^{tot}-E_{c}^{tot}$ is the change of the total energy (longitudinally corrected in the case of $E_{c}^{tot}$ ) upon adding one electron to the ionic system. The latter is expressed as

8 $$\Delta E_{c}^{tot}=-e\Phi_{0}+E_{s}$$

where *E*_*s*_ is the energy arising from the finite surface dipole formed upon neutral adsorption. Using the calculated values of $\Delta E_{c}^{tot}$ and Φ_0_ = 4.24 V, we find *E*_*s*_ = 1.63, 0.90, and 0.50 eV, respectively, for the 3 × 2, 4 × 3, and 6 × 4 cells. At infinite cell size, the surface dipole (∝1/Ω) vanishes, implying, from eqs 7 and 8,

9 $$E_{0}\left(z_{ads}\right)=-e\Phi_{0}+E\left(z_{ads}\right)+IE$$

With calculated ionization energies of 5.48, 5.22, and 4.39 eV, we obtain OBC adsorption energies of −1.77, −1.22 and −1.62 eV for Li^0^, Na^0^ and K^0^, respectively. In contrast to the ionic case and as noted by Valencia al.,^30^ the trend is no longer monotonic vs the atomic number because of the specific counter-effect of the ionization energy.

Beyond the case of having an entire charge on a monoatomic ion and facing a metallic surface, as illustrated by the previous systems, the present correction approach can be generalized to more complex ionic systems and surfaces of finite permittivity, including semiconductors. For this, the expression of eq 5 should be replaced by the Ewald energy of a multicentered charge distribution facing a dielectric slab, akin to the one derived in ref 2 for a screening medium of finite permittivity. Multicentered charge distributions include the case of molecular ions but also of partial charge transfers upon chemisorption, as frequently encountered in electrocatalysis. In all cases, the sensitivity of the energy correction to the selected charge partition scheme should be evaluated. Such generalized applications of the method could be addressed in follow-up studies.

## Conclusion

An analytical scheme has been proposed to correct a posteriori the energies of charged adsorbates on metallic surfaces from periodic-boundary to open-boundary conditions. The parameter *z*_1_ of the underlying metal surrogate model can be estimated from fitting error minimization or directly from the charging behavior of the surface. In the latter case, the correction procedure then requires, in principle, the PBC adsorption profile from only one cell size. The accuracy of the analytical correction tends to degrade at short distances (here, typically, shorter than the adsorption position), then calling for a more refined electrostatic model as performed numerically for embedded charge defects.^6–8^

The method is illustrated by determining the OBC adsorption energies of ionic and neutral lithium, sodium and potassium on graphite from first principles. In this case, it also helped clarify the ionic binding mechanism as being mainly electrostatic, namely, the sum of the image-charge interaction and a short-range, repulsive electrostatic interaction.

Capacitively corrected ion adsorption energy profiles are shown to converge slowly with the simulation cell size, with typical differences of the order of 1 eV between the 6 × 3 and infinite cells, emphasizing the importance of the present lateral correction scheme to recover the appropriate infinite-cell limit.

## Data Availability

A typical input file to obtain the calculated energies of Figure 4 is available on Materials Cloud (DOI:10.24435/materialscloud:1n-sa).

## References

[1] Makov G., Payne M. C. (1995). Periodic boundary conditions in ab initio calculations. Phys. Rev. B.

[2] Otani M., Sugino O. (2006). First-principles calculations of
charged surfaces and
interfaces: A plane-wave non-repeated slab approach. Phys. Rev. B.

[3] Dabo I., Kozinsky B., Singh-Miller N., Marzari N. (2008). Electrostatics in periodic
boundary conditions and real-space corrections. Phys. Rev. B.

[4] Andreussi O., Marzari N. (2014). Electrostatics of solvated
systems in periodic boundary
conditions. Phys. Rev. B.

[5] Freysoldt C., Neugebauer J., Tan A. M. Z., Hennig R. G. (2022). Limitations
of empirical
supercell extrapolation for calculations of point defects in bulk,
at surfaces, and in two-dimensional materials. Phys. Rev. B.

[6] Komsa H.-P., Pasquarello A. (2013). Finite-size
supercell correction for charged defects
at surfaces and interfaces. Phys. Rev. Lett..

[7] Komsa H.-P., Berseneva N., Krasheninnikov A. V., Nieminem R. M. (2014). Charged point defects
in the flatland: Accurate formation energy calculations in two-dimensional
materials. Phys. Rev. X.

[8] Freysoldt C., Neugebauer J. (2018). First-principles calculations for charged defects at
surfaces, interfaces, and two-dimensional materials in the presence
of electric fields. Phys. Rev. B.

[9] Durrant T. R., Murphy S. T., Watkins M. B., Shluger A. L. (2018). Relation between
image charge and potential alignment corrections for charged defects
in periodic boundary conditions. J. Chem. Phys..

[10] Rozzi C. A., Varsano D., Marini A., Gross E. K. U., Rubio A. (2006). Exact Coulomb
cutoff technique for supercell calculations. Phys. Rev. B.

[11] Ismail-Beigi S. (2006). Truncation
of periodic image interactions for confined systems. Phys. Rev. B.

[12] Vijay, S. ; Schlipf, M. ; Miranda, H. ; Karsai, F. ; Kaltak, M. ; Marsman, M. ; Kresse, G. Efficient periodic density functional theory calculations of charged molecules and surfaces using Coulomb kernel truncation. arXiv. 2006, 10.48550/arXiv.2501.02435. (accessed 2025–05–01).

[13] Da
Silva M. C., Lorke M., Aradi B., Tabriz M. F., Frauenheim T., Rubio A., Rocca D., Deak P. (2021). Self-consistent
potential correction for charged periodic systems. Phys. Rev. Lett..

[14] Kumagai Y. (2024). Corrections
on formation energies and eigenvalues of point defect calculations
in two-dimensional materials. Phys. Rev. B.

[15] Chan K., Nørskov J. K. (2015). Electrochemical
barriers made simple. J. Phys. Chem. Lett..

[16] Chan K., Nørskov J. K. (2016). Potential
dependence of electrochemical barriers from
ab initio calculations. J. Phys. Chem. Lett..

[17] Castleton C. W. M., Höglund A., Mirbt S. (2006). Managing the supercell
approximation for charged defects in semiconductors: Finite-size scaling,
charge correction factors, the band-gap problem, and the ab initio
dielectric constant. Phys. Rev. B.

[18] Wright A. F., Modine N. A. (2006). Comparison of two
methods for circumventing the Coulomb
divergence in supercell calculations for charged point defects. Phys. Rev. B.

[19] Shim J., Lee E.-K., Nieminen R. M. (2005). Density-functional
calculations of
defect formation energies using supercell methods: Defects in diamond. Phys. Rev. B.

[20] Andreussi O., Dabo I., Marzari N. (2012). Revised self-consistent
continuum
solvation in electronic-structure calculations. J. Chem. Phys..

[21] A module to handle environment effects in first-principles condensed-matter simulations, http://www.quantum-environ.org/. (accessed February 6, 2025).

[22] Prajapati A. K., Bhatnagar A. (2023). A review on anode materials for lithium/sodium-ion
batteries. J. Ener. Chem..

[23] Schmickler, W. ; Santos, E. Interfacial Electrochemistry; Springer, 2010.

[24] Beinlich S.
D., Kaslunger G., Reuter K., Hörmann N. G. (2023). Controlled
electrochemical barrier calculations without potential control. J. Chem. Theory Comput..

[25] Hörmann N. G., Beinlich S. D., Reuter K. (2024). Converging divergent
paths: Constant
charge vs constant potential energetics in computational electrochemistry. J. Phys. Chem. C.

[26] Quantum ESPRESSO, https://www.quantum-espresso.org/. (accessed 29 April 2024).

[27] Perdew J. P., Burke K., Ernzerhof M. (1996). Generalized gradient approximation
made simple. Phys. Rev. Lett..

[28] A standard solid-state pseudopotentials (SSSP) library optimized for precision or efficiency, https://www.materialscloud.org/discover/sssp/table/efficiency. (accessed 29 April 2024).

[29] Santos E. J. G., Kaxiras E. (2013). Electric-field dependence of the effective dielectric
constant in graphene. Nano Lett..

[30] Valencia F., Romero A. H., Ancilotto F., Silvestrelli P. L. (2006). Lithium
adsorption on graphite from density functional theory calculations. J. Phys. Chem. B.

[31] Garay-Tapia A. M., Romero A. H., Barone V. (2012). Lithium adsorption on graphene: From
isolated adatoms to metallic sheets. J. Chem.
Theory Comp..

